# Climate Change Reduces Habitat Suitability of the Endemic Iranian Ground‐Jay (*Podoces pleskei*): Spatial Analyses to Guide Conservation Strategies

**DOI:** 10.1002/ece3.73637

**Published:** 2026-06-09

**Authors:** Masoud Yousefi, Bagher Nezami, Faham Asgari, Sayyad Sheykhi Illanloo, Sayedeh Alemohammad, Luciano Bosso

**Affiliations:** ^1^ Faculty of Governance University of Tehran Tehran Iran; ^2^ Research Group of Biodiversity & Biosafety Research Center for Environment and Sustainable Development Tehran Iran; ^3^ Department of Environmental Science Gorgan University of Agricultural Sciences and Natural Resources Gorgan Iran; ^4^ Institute for Agriculture and Forestry Systems in the Mediterranean National Research Council of Italy Portici Italy

**Keywords:** climate change, conservation planning, dryland ecosystems, ecological niche model, habitat suitability, protected areas

## Abstract

Dryland and semiarid ecosystems in Iran are increasingly threatened by climate change and human activities, posing significant risks to endemic avian species such as the Iranian Ground‐jay ( *Podoces pleskei* ). In this study, we used ecological niche models and GIS analyses to predict the impacts of climate change on the habitat suitability of this bird. Our models showed that the habitat suitability for *P. pleskei* was primarily concentrated within the deserts and xeric shrublands biomes and identified extensive suitable habitat patches along the Iran–Pakistan border. *Podoces pleskei* showed a higher probability of presence at low values of mean temperature of the driest quarter, annual precipitation, NDVI, and distance from human settlements. Our findings reveal that although approximately 36% of suitable habitats fall within formally designated protected areas, the majority remain unprotected and suffer from severe fragmentation, compromising long‐term conservation prospects. We found that *P. pleskei* lost a considerable proportion of its suitable habitats due to climate change i.e., 18% and 52% under SSP1‐2.6 and SSP5‐8.5 scenarios, respectively. Our models showed strong predictive performance as indicated by high Area Under Curve and True Skill Statistic values. Moving beyond traditional protected area designation, conservation efforts must prioritize habitat connectivity and community engagement to ensure the persistence of *P. pleskei* and other dryland avian species, particularly in light of the significant habitat loss caused by climate change. Our findings contribute valuable insights into avian ecology in Iran's fragile ecosystems and inform evidence‐based conservation planning in the country.

## Graphical abstract

Pleske's ground jay ( *Podoces pleskei* ), an endemic bird of Iran's drylands, faces severe habitat loss due to climate change and human activities. Using ecological niche models and GIS analyses, we identified key environmental drivers of its distribution and found that 52% of suitable habitat is projected to be lost, with much of the remaining range unprotected and fragmented. Our results highlight the need for habitat connectivity and community‐based conservation strategies to ensure the species' long‐term persistence.

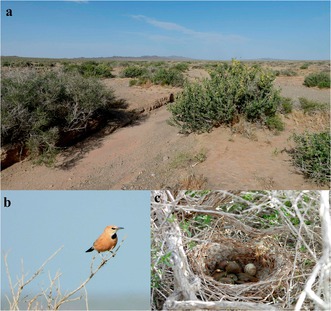

## 1 Introduction

Climate change is a major global threat to all species on our planet (Hannah 2021; Mahecha et al. 2024; Panero et al. 2026). Animal species are especially sensitive to its effects, as it alters their communities, geographic distributions, and phenology (Pacifici et al. 2020; Mahecha et al. 2024; Ahmad et al. 2025). The naïve model of shifts in distribution under climate change is that species will usually move poleward in latitude and upward in elevation (Hannah 2021). Indeed, in many instances, recent upward/poleward shifts have been attributed to climate change (La Sorte and Thompson 2007; Parolo and Rossi 2008; Lenoir and Svenning 2015; Wu 2015). The potential impacts of climate change on avian species are rarely documented in Iran, and only a handful of studies predicted range expansions and contractions, as well as eastward shifts in avian distributions in the country, including *Dendrocopos major* , *Phasianus colchicus* , *Chlamydotis macqueenii* , and *Gypaetus barbatus* (Yousefi et al. 2017; Sheykhi Ilanloo et al. 2021; Asgharzadeh et al. 2023; Sheykhi et al. 2025). To predict future distributions and to assess the impacts of climate change, Ecological Niche Models (ENMs) integrated with GIS‐based analyses provide robust and widely adopted tools (Anderson 2013; Di Febbraro et al. 2023).

ENMs have become a cornerstone in avian research, with applications spanning ecology, conservation, evolution, and taxonomy (Engler et al. 2017; Yousefi et al. 2025). In ornithological studies, ENMs have been widely used to model habitat suitability across multiple spatial scales, from local landscapes to global extents (Vignali et al. 2021; Bai et al. 2022). These approaches have proven particularly valuable for identifying conservation priorities and biodiversity hotspots (Moradi et al. 2019; Buechley et al. 2022), forecasting the effects of climate change on bird distributions (Zhu et al. 2022; Escobar‐Luján et al. 2022) and evaluating the effectiveness of protected areas in safeguarding avian populations (Tian et al. 2022; Buechley et al. 2022). However, this approach is still little used in Iranian conservation studies, for instance, the Asian Houbara Bustard, *Chlamydotis macqueenii* (Yousefi et al. 2017); the little owl, *Athene noctua* (Sheykhi Ilanloo et al. 2020); the bearded vulture, *Gypaetus barbatus* (Sheykhi Ilanloo et al. 2021); and the common pheasant, *Phasianus colchicus* (Ashoori et al. 2018; Asgharzadeh et al. 2023). Iran is a large country located in Southwest Asia, which is known for its high biological diversity due to its diverse topography and rich historical events like mountain uplifting and climatic fluctuations, as well as many geographical barriers (reviewed in Yousefi et al. 2023). However, its biodiversity, especially birds, is threatened by habitat loss, environmental pollution, land use change, climate change, and hunting (Ashoori et al. 2018; Sheykhi Ilanloo et al. 2021; Asgharzadeh et al. 2023). With 559 species, birds are the most species‐rich group of Iranian vertebrates, but they have fewer endemic species than other vertebrate groups (Scott et al. 1975; Mansoori 2008; Yousefi et al. 2023). In fact, the only endemic bird species is the Iranian Ground‐jay ( *Podoces pleskei* ) (Scott et al. 1975; Mansoori 2008).

*Podoces pleskei* is known from the arid and semiarid ecosystems of the Central Iranian Plateau (Scott et al. 1975; Mansoori 2008). The species occurrence matches the deserts and xeric shrublands biome in Iran (Dinerstein et al. 2017). The population trend of the species appears to be stable, but the population size has not been quantified (BirdLife International 2024). The species' conservation status was reported to be Near Threatened in 1988, then its status changed to Lower Risk/Least Concern in 1994, and from 2004 onward, it has been known as Least Concern (BirdLife International 2024). The extent of occurrence (breeding/resident) is estimated to be around 475,000 km^2^ (BirdLife International 2024). Although this species is the only endemic bird species in Iran, its distribution patterns and habitat preferences remain largely unknown, particularly at a large spatial scale (Scott et al. 1975; Mansoori 2008; Alizadeh et al. 2021; BirdLife International 2024). Previous studies have indicated that ENMs can increase our understanding of avian ecology and conservation in Iran; however, to the best of our knowledge, this work represents the first attempt to apply such an approach to endemic *P. pleskei* throughout the species' entire distribution range. Endemic species are especially vulnerable to climate change because of having a smaller distribution range and smaller population size compared to other species (Yousefi et al. 2020, 2024; Manes et al. 2021). Thus, it is important to study and quantify the impacts of climate change on these unique elements of biodiversity in each country.

This study aims to identify the most suitable habitats of *P. pleskei* , determine the key environmental drivers shaping its distribution, assess the coverage of suitable habitats within Iran's protected areas network, and predict potential shifts in the species' distribution under future climate change scenarios. Given that *P. pleskei* is associated with arid and semiarid ecosystems, we hypothesized that, as observed for other birds of arid environments, topographic features would represent the primary determinants of its distribution across Iran's arid landscapes (Carrascal et al. 2008). Furthermore, considering the limited development of protected areas within Iran's arid ecosystems (Darvishsefat 2008), we expected that a substantial proportion of suitable habitats of this endemic bird would fall outside the existing protected areas network, where they are more likely to be exposed to human disturbance.

## 2 Materials and Methods

### 2.1 Study Area

Iran is a large country covering approximately 1,648,195 km^2^. Annual mean precipitation ranges from less than 50 mm to over 2000 mm, while annual mean temperatures vary between 7.1°C and 27.6°C across the country (Kafash et al. 2020). The Alborz, Zagros, and Kopet‐Dagh mountains are the major mountain chains in the country, hosting high species diversity and richness (Yousefi et al. 2023). The country's pronounced climatic heterogeneity and high topographic diversity support a wide diversity of habitats, including mangrove ecosystems along the southern coasts, extensive sand dunes in the central deserts, and alpine meadows and temperate deciduous broad‐leaved forests in the north (Darvishsefat 2008; Dinerstein et al. 2017). Iran also hosts 84 wetlands of international importance, 33 of which are designated as Ramsar sites (Aazami and Joorabian Shooshtari 2023). Together, these natural and human‐made ecosystems play a crucial role in the conservation of avian diversity and provide essential stopover sites for migratory bird species (Khani et al. 2015).

### 2.2 Occurrence Data

**FIGURE 1:**
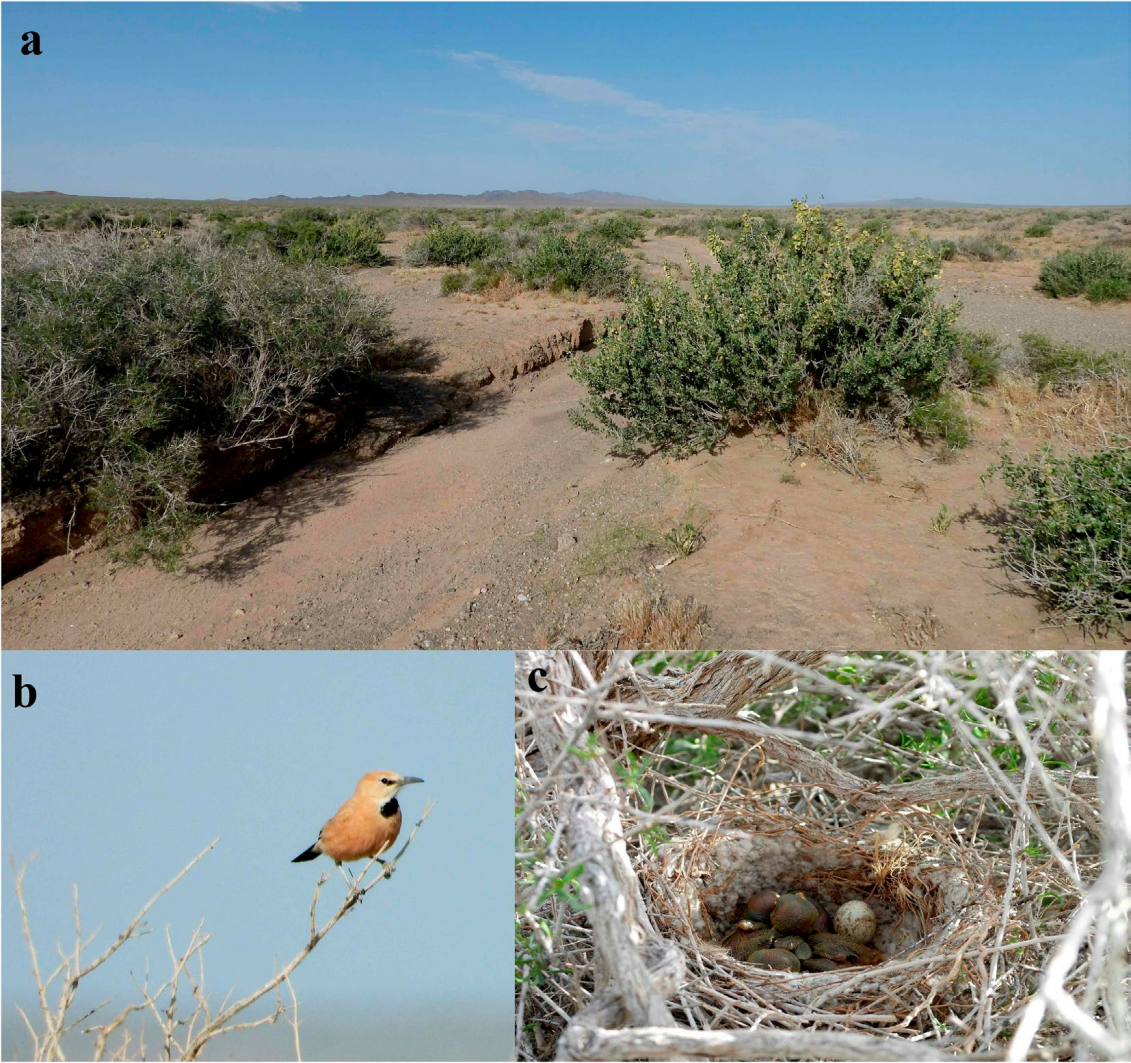
Breeding habitat (a), bird (b), and the nest with egg and chicks (c) of *Podoces pleskei* in the Touran Biosphere Reserve. Photographs provided by Masoud Yousefi.

We obtained occurrence records of *P. pleskei* (Figure 1) from multiple sources, including online databases such as Global Biodiversity Information Facility (https://www.gbif.org/, GBIF 2024), eBird (https://ebird.org/) and VertNet (https://www.vertnet.org/), as well as from our own opportunistic field observations collected between 2008 and 2023 across the species' entire distribution range in Iran. Records from online databases spanned the period from 2000 to 2022, with the majority originating from the eBird database. In total, we compiled 245 presence records. We removed duplicate records and applied spatial thinning using a minimum distance of 1 km to match the resolution of the environmental variables (e.g., Bosso et al. 2026). After data filtering (removal of 14 duplicate records and 22 records through spatial thinning), the final dataset consisted of 111 occurrence records used for ENMs (Table S1; Kafash 2024).

### 2.3 Environmental Variables

To characterize the environmental conditions of *P. pleskei* 's habitat, we selected environmental variables related to climate, topography, vegetation, and human presence (Moosavinezhad 2012; Radnezhad et al. 2013; Yousefi et al. 2017; Alizadeh et al. 2021). We obtained climatic variables, including mean temperature of the driest quarter, annual precipitation, and precipitation of the driest quarter, from the CHELSA database (http://chelsa‐climate.org/), which provides high‐resolution climate data at 30 arcsec (~1 km at the equator) (Karger et al. 2017). We derived elevation data from the Shuttle Radar Topography Mission (SRTM) digital elevation model, which was downloaded from the EarthEnv (http://www.earthenv.org/), and we calculated slope using the terrain function in the ‘*raster*’ package (Hijmans et al. 2015) in R (R Core Team 2023). We considered vegetation as a key factor shaping species distributions in arid and semiarid ecosystems; therefore, we included the Normalized Difference Vegetation Index (NDVI) and distance to shrublands to assess their influence on the species' distribution. To account for human presence, we included distance to human settlements and distance to roads (Sheykhi Ilanloo et al. 2021). To this end, Euclidean distance to the nearest cover patch of shrublands, human settlements, and roads was calculated. We resampled all nonclimatic variables to match the spatial resolution of the climatic grids (1 × 1 km). To reduce multicollinearity among predictors, we calculated Variance Inflation Factors (VIF; Quinn and Keough 2002) using the ‘*usdm*’ package (Naimi 2015), which showed low collinearity among the selected variables (Table 1; Guisan et al. 2017). We selected all the variables with a VIF < 5.

**TABLE 1:** Environmental variables used to detect current habitat suitability of *Podoces pleskei* .

Type	Variable	VIF value
Topography	Elevation	1.488
	Slope	1.601
Vegetation	Distance from shrub‐lands	2.185
	NDVI	2.842
Anthropogenic	Distance from human settlement	1.885
	Distance from road	2.731
Climate	Mean temperature of driest quarter	2.280
	Annual precipitation	2.492
	Precipitation of driest quarter	3.453

### 2.4 Ecological Niche Models

To model the habitat suitability of *P. pleskei* , we used the ‘*sdm’* package (Naimi and Araújo 2016) in the R environment v. 3.1.2 (R Core Team 2023) to fit SDMs using five algorithms: Generalized Linear Models (GLM, McCullagh and Nelder 1989), Generalized Additive Models (GAM, Hastie and Tibshirani 1990), Generalized Boosting Models (GBM, Ridgeway 1999), Maximum Entropy (Maxent, Phillips et al. 2006) and Random Forest (RF, Breiman 2001). We then applied an ensemble approach (Araújo and New 2007) to combine the predictions generated by different individual SDMs into a single final distribution (e.g., Fida et al. 2025). Considering that these methods need pseudo‐absences. We generated 5000 pseudo‐absences by randomly sampling coordinates from the deserts and xeric shrublands biomes where the species occurred. The models were developed using the randomly selected 80% of the records for training and 20% of the data for tests. We converted the species' continuous habitat suitability map into a binary (suitable/unsuitable) map using the sensitivity–specificity sum maximization method in the R package ‘*Presence/Absence* 1.1.9’ (Freeman and Moisen 2008).

To assess the predictive performance of the models, we used the area under the receiver operating characteristic curve (AUC; Fielding and Bell 1997) and the True Skill Statistic (TSS). The AUC values range from 0 to 1, where values closer to 1 indicate a higher accuracy of model projection (Fielding and Bell 1997). TSS values range between −1 and + 1. A TSS value of +1 means complete agreement between observed and projected distributions, whereas values of ≤ 0 denote no better than random performance (Allouche et al. 2006).

### 2.5 Conservation Gap Analysis

We estimated the coverage of protected areas overlapping the suitable habitats of *P. pleskei* in QGIS 3.38.1 (QGIS.org 2026). We downloaded the protected area in Iran from the Department of Environment of Iran (2022; Figure S1).

### 2.6 Climate Change Projections

In order to predict the impacts of climate change on the habitat suitability, we downloaded the bioclimatic variables from CHELSA (Karger et al. 2017) at a 30 arcsec (~1 km at the equator) resolution for current climate and future climate (year 2071–2100) scenarios from five different CMIP6 Global Circulation Models (GCMs), such as GFDL‐ESM4, IPSLCM6A‐LR, MPI‐ESM1‐2‐HR, MR‐ESM2‐0, UKESM1‐0‐LL. CMIP6 GCMs are state‐of‐the‐art, and using several GCMs is reducing the uncertainty coming from the single GCMs. To use for future projection, we averaged these five GCMs and produced a single layer for each climatic variable. Further, we used two different Shared Socioeconomic Pathways (SSPs), such as SSP1‐2.6 and SSP5‐8.5. We considered, after VIF analysis, seven climatic variables in projecting future habitat suitability of *P. pleskei* (Table 2) and used the same modeling approach described in the ENMs paragraph.

**TABLE 2:** Climatic variables used to detect the habitat suitability of *Podoces pleskei* in the future scenarios.

Variable	VIF value
Annual mean temperature	5.139
Isothermality	1.841
Temperature annual range	1.832
Mean temperature of wettest quarter	3.831
Annual precipitation	2.021
Precipitation of driest month	3.059
Precipitation seasonality	3.112

## 3 Results

### 3.1 Current Model and Conservation Gap Analysis

**FIGURE 2:**
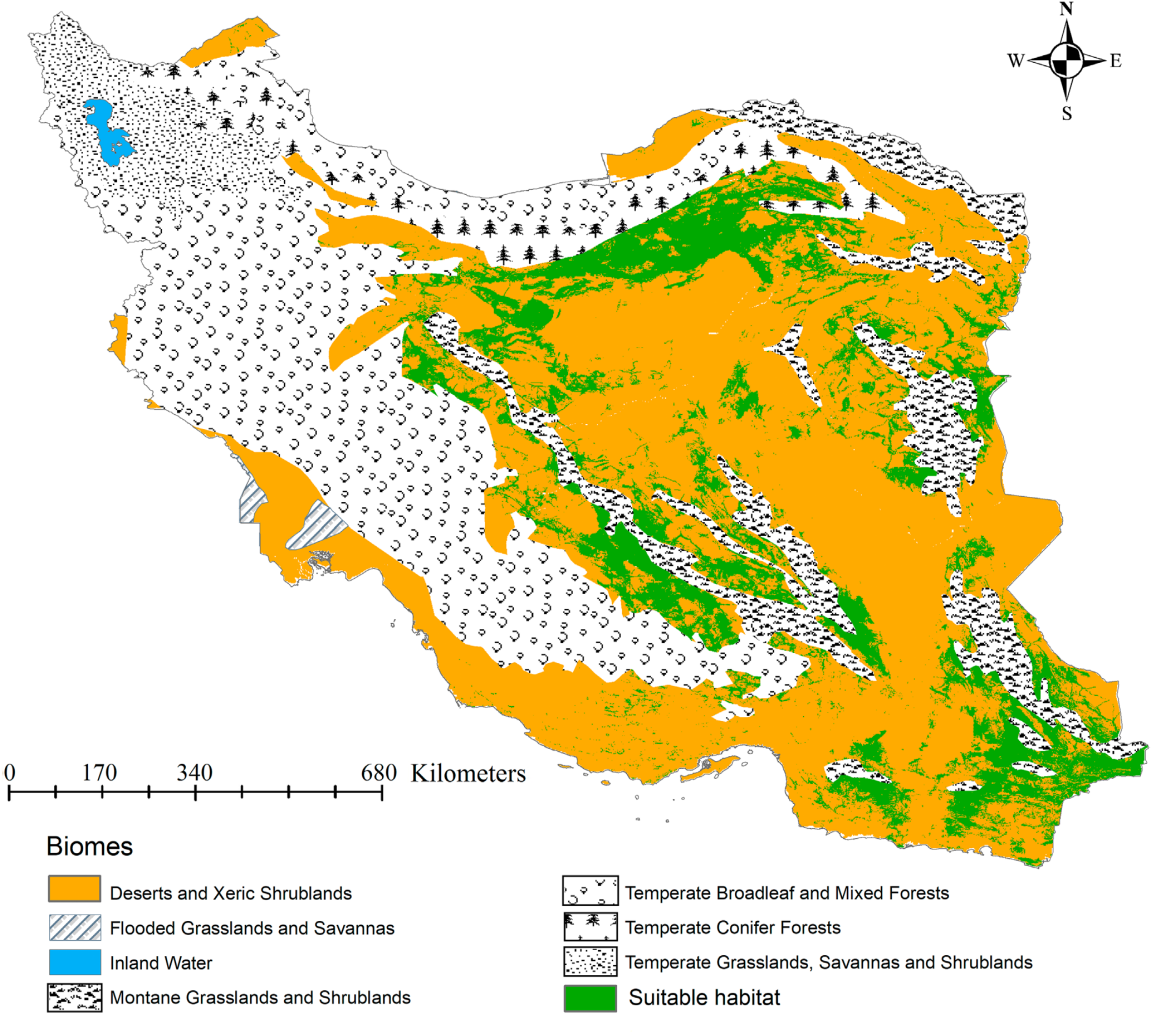
Iranian biomes and habitat suitability map of *Podoces pleskei* .

**FIGURE 3:**
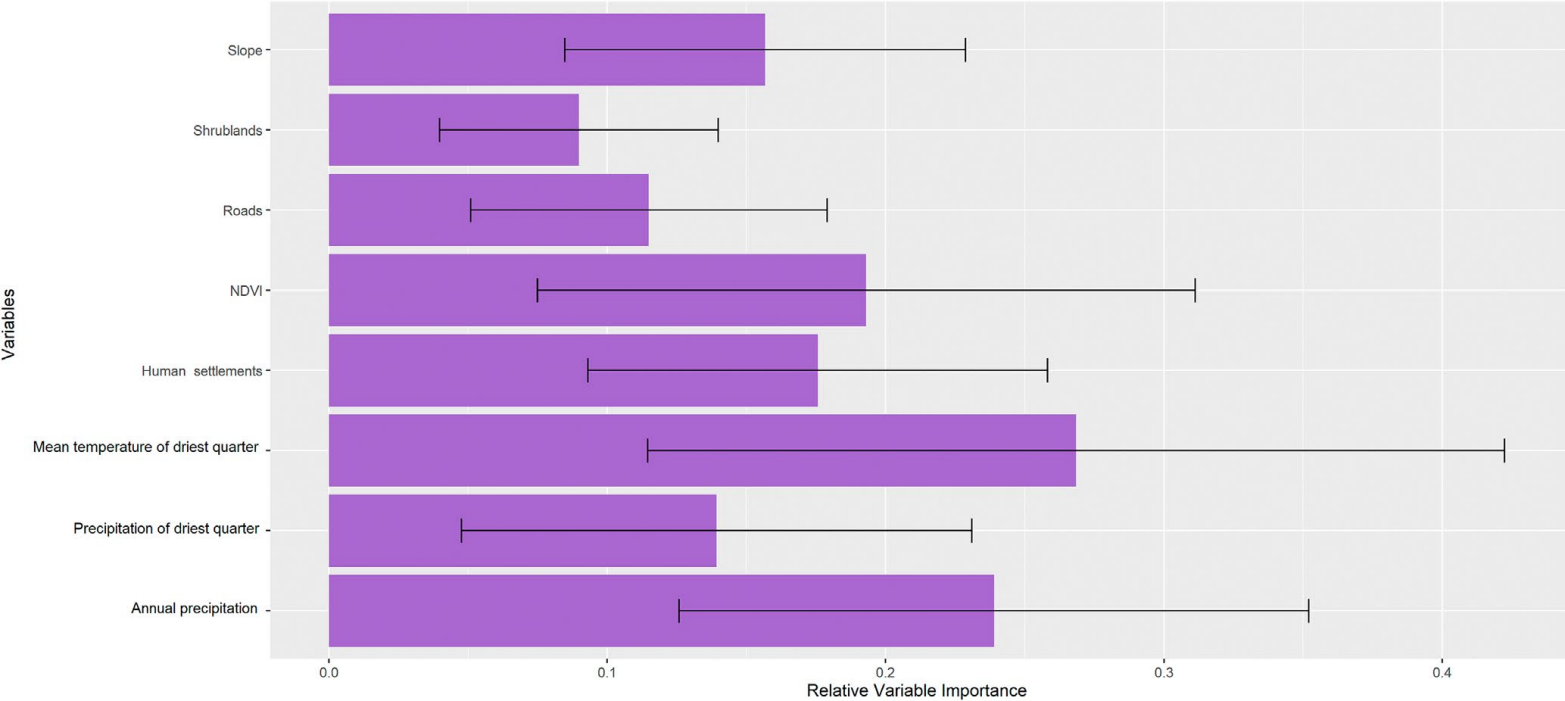
Variable importance in shaping habitat suitability of *Podoces pleskei* .

Our models showed strong predictive performance, as indicated by high AUC (GLM = 0.93, GAM = 0.89, GBM = 0.94, Maxent = 0.95 and RF = 0.94) and TSS values (GLM = 0.79, GAM = 0.76, GBM = 0.83, Maxent = 0.85 and RF = 0.83). We found that the habitat suitability for *P. pleskei* was primarily concentrated within the desert and xeric shrublands biome with a highly fragmented and heterogeneous pattern across the study area (Figure 3). The largest and most continuous suitable habitat patches occurred south of the temperate conifer forest biome, while the montane grasslands and shrublands spatially overlapped with and surrounded most of the other suitable areas. Notably, the model identified extensive suitable habitat patches along the Iran–Pakistan border (Figure 2).

**FIGURE 4:**
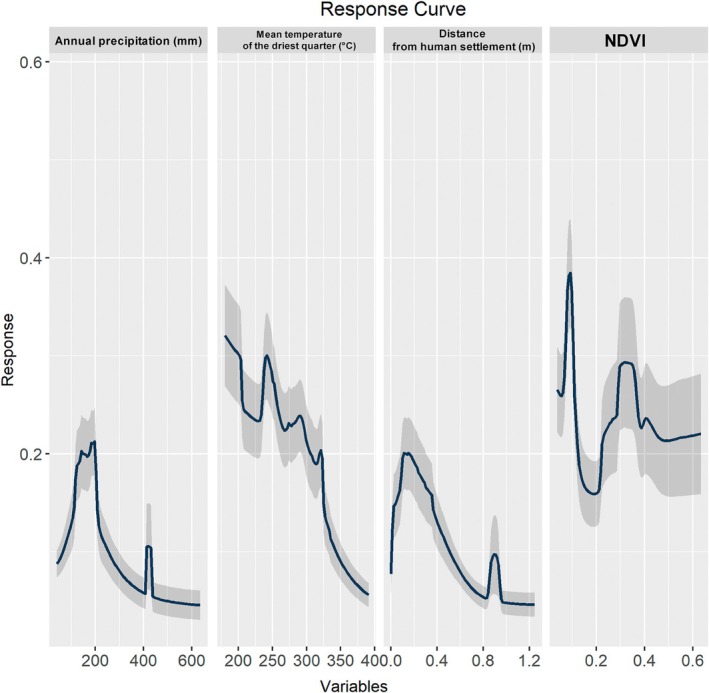
Response curves of the most important environmental variables in shaping *Podoces pleskei* .

We found that the mean temperature of the driest quarter was the most influential factor determining the species' distribution, followed by annual precipitation, NDVI, and human settlement density (Figure 3). *Podoces pleskei* showed higher probabilities of presence at low values of temperatures of the driest quarter, annual precipitation, NDVI, and distance from human settlements (Figure 4).

**FIGURE 5:**
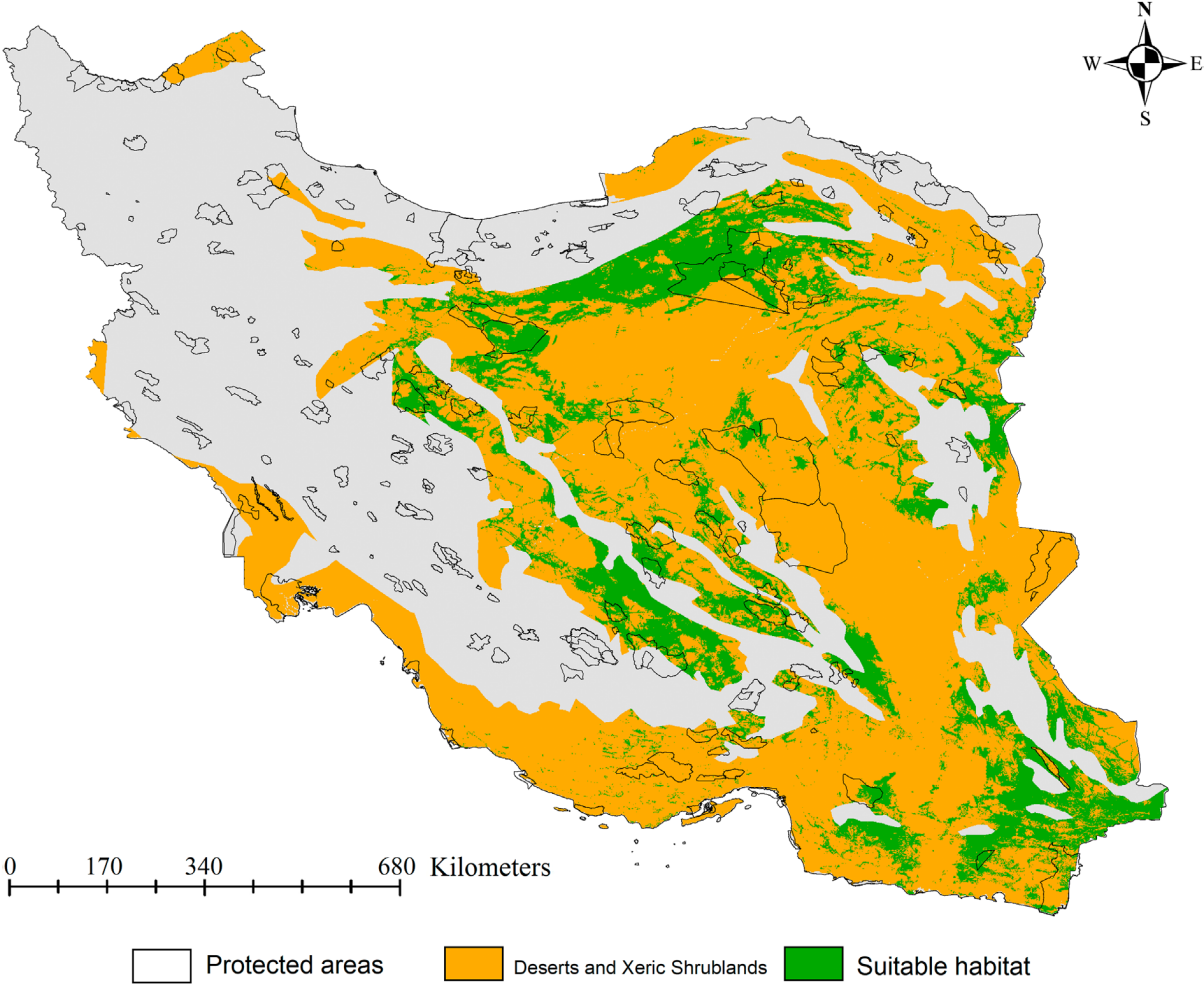
*Podoces pleskei* 's suitable habitat, Iran's protected areas and Iranian desert and xeric shrubland biome.

Finally, our map showed that 36% of the suitable habitat of *P. pleskei* (Figure 5) fell within the protected areas. While some key habitat patches fall within protected boundaries—particularly in central and northern mountainous regions—a substantial proportion of suitable habitat remains outside protected areas.

### 3.2 Climate Change Models

**FIGURE 6:**
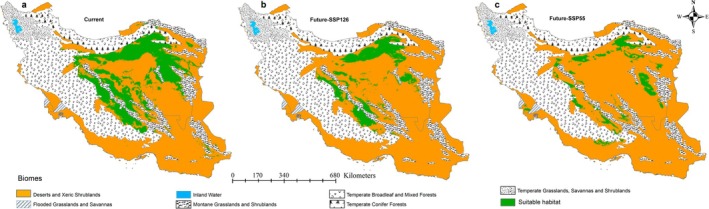
Climate change models for *Podoces pleskei* in Iran based on the mean of five different CMIP6 Global Circulation Models such as GFDL‐ESM4, IPSLCM6A‐LR, MPI‐ESM1‐2‐HR, MR‐ESM2‐0, UKESM1‐0‐LL and under three scenarios: Current (a), SSP1‐2.6 (b), and SSP5‐8.5 (c).

We found the climate change models performed well according to AUC (GLM = 0.78, GAM = 0.09, GBM = 0.89, Maxent = 0.82, and RF = 0.91) and TSS (GLM = 0.62, GAM = 0.78, GBM = 0.79, Maxent = 0.66, and RF = 0.82) values. We predicted future distribution of *P. pleskei* under the two different climate change scenarios (Figure 6). We found that the species' suitable habitat will decrease by 18% under SSP1‐2.6 and 52% under SSP5‐8.5. Our results showed that annual mean temperature (32% contribution), annual precipitation (26.5%) and temperature annual range (18%) were the most important climatic variables in shaping the species distribution.

## 4 Discussion

Endemic species warrant particular attention because they represent unique evolutionary lineages (e.g., Panero et al. 2026). In this study, we identified the most suitable areas for *P. pleskei* and detected a substantial decline in suitable habitat under future climate change scenarios.

### 4.1 Current and Future Habitat Suitability of *P. pleskei*

The largest suitable habitat patches were located north of the deserts and xeric shrublands biome and south of the Central Iranian Plateau, highlighting these regions as high‐priority areas for conservation planning. Although no previous habitat suitability maps are available for direct comparison, our results are consistent with existing knowledge of the species' distribution (Scott et al. 1975; Mansoori 2008). But our model shows a wider distribution range for this species. It should be noted that *P. pleskei* is known to be endemic to Iran (Scott et al. 1975; Mansoori 2008; Birdlife International 2024); however, our habitat suitability model showed large suitable patches along the Iran and Pakistan border. This implies that it can also be found in Pakistan, at least in areas close to the Iran border. We encourage targeted field surveys by ornithologists and experienced birdwatchers in potentially suitable habitats along the Iran–Pakistan border to confirm whether *P. pleskei* occurs in Pakistan.

We initially expected topography, particularly slope, to be the most important variable in shaping this bird in Iran; however, climatic variables (mean temperature of the driest quarter and annual precipitation) were found to be the most influential determinants of the species distribution. We think it is the high climatic variability within the country that makes climatic factors predominant determinants of biodiversity distribution in Iran (Scott et al. 1975; Kafash et al. 2020; Yousefi et al. 2023). Our results indicate that slope is the fifth most important factor for the species. *Podoces pleskei* largely occurs with the Asian Houbara Bustard and that suitability is reduced in regions with slopes > 10%. Consequently, major mountainous areas in the country fall into the range of unsuitable habitats in our model. *Podoces pleskei* prefers plain areas up to lands with moderate slopes (Hamedanian 1997; Moosavinezhad 2012; Mohammadi et al. 2015). This type of habitat in desert areas is suitable for the behavior of short flights at an elevation close to the ground and sitting on the ground with a quick run among the bushes (Rasouli et al. 2017).

We found that NDVI is the third most important environmental predictor of *P. pleskei* . The value close to zero showed that the absence of vegetation is a very important factor for the distribution of the species across the arid and semiarid ecosystems of Iran. Confirming our results, Mohammadi et al. (2015) showed that the presence of *Zygophyllum eurypterus* is very critical for the distribution of the species, particularly during the breeding season. According to Khaleghizadeh and Sehhatisabet (2006), *P. pleskei* eats some parts of roots and stems of desert plants such as *Zygophyllum* spp. and *Tamarix* spp., which grow after rainfall.

Roads are an important form of human development, and the transport networks associated with them have long impacted wildlife and habitats (Forman and Alexander 1998; Trombulak and Frissell 2000; Benítez‐López et al. 2010; Rehan et al. 2024). But in this study, we found that at large scale, distance to roads variable did not play an important role in shaping the species distribution. However, field observations and previous studies have shown that in some areas, this species tends to be present around roads (Alizadeh et al. 2021). The reason for the presence of this bird around roads is probably the availability of food, such as food remnants from cars and trucks, and the presence of insects on the carcasses of dead animals on the roads (Londei 2011; Alizadeh et al. 2021). We further recommend multiscale studies to better understand the species ecology and disentangle the influence of environmental factors on the species distribution (Rose et al. 2020; Redlich et al. 2022; Suárez‐Castro et al. 2022).

Climate change is an unavoidable threat for biodiversity worldwide, and its negative impacts are increasing at an unprecedented rate (Lawler et al. 2009; Chen et al. 2011; Bellard et al. 2012; Thuiller et al. 2019; Fialas et al. 2025). Previous studies have demonstrated that climate change represents a major threat to biodiversity in Iran (reviewed in Yousefi et al. 2019). Here, several bird species are already predicted to experience significant reductions in suitable habitat under future climate scenarios, including *Dendrocopos major* , *Phasianus colchicus* , *Chlamydotis macqueenii* , and *Gypaetus barbatus* (Yousefi et al. 2017; Sheykhi Ilanloo et al. 2021; Asgharzadeh et al. 2023; Sheykhi et al. 2025). Desert and semidesert specialists appear to be particularly vulnerable, as their distributions are tightly linked to narrow climatic conditions and low‐productivity ecosystems. In line with these findings, our results indicate that *P. pleskei* is also likely to undergo a severe decline in suitable habitat in the future. Consequently, we propose that the remaining suitable areas should be considered high‐priority targets for conservation planning. This pattern is consistent with broader global assessments showing that climate change is driving widespread declines in bird populations and substantial shifts in species distributions, particularly in biodiversity hotspots where endemic taxa are inherently range‐restricted and have limited capacity to track rapid environmental change (Huntley et al. 2008; Şekercioğlu et al. 2012).

### 4.2 Conservation Gap Analysis

Protected areas are key instruments for biodiversity conservation (Leverington et al. 2010; Watson et al. 2014; Gray et al. 2016). Assessing the extent to which suitable habitats are represented within existing protected area networks is essential to identify conservation gaps, guide future expansions, and evaluate their effectiveness in achieving biodiversity objectives (Yousefi et al. 2022; Zeng et al. 2023). Here we showed that only 36% of the suitable habitat of *P. pleskei* is located within the existing protected area network. In comparison, 64% of the suitable habitats have no legal protection. On the other hand, this means that most of the species' habitat faces human disturbances like overgrazing, road development, mining, etc.

Our nationwide habitat suitability modeling for *P. pleskei* reveals a highly fragmented pattern of suitable habitats, largely confined to central and eastern deserts and semidesert plateaus. Interestingly, a substantial proportion of these high‐suitability areas lie outside the current network of protected areas. This spatial mismatch suggests that existing conservation planning may not fully capture the ecological requirements of this endemic species, which depends on sparse shrubland and desert steppe habitats that are often overlooked in national reserve design. The identification of these unprotected yet ecologically important areas creates a clear opportunity for conservation managers to reassess and potentially expand protected area boundaries or to implement targeted habitat restoration and community‐based conservation measures. By integrating fine‐scale habitat suitability data into national conservation strategies, we can better safeguard this flagship desert species and, by extension, the fragile desert ecosystems it represents. In addition, this study identified areas that will remain suitable under future climate change, which will play an important role for the species' conservation under the changing climate. These stable suitable habitats have high priority for long‐term persistence and conservation.

## 5 Conclusions

In this study, we developed the first country‐wide high‐resolution habitat suitability model for *P. pleskei* , the only endemic bird species in Iran (Scott et al. 1975; Yousefi et al. 2023) and identified its most suitable habitat. This endemic bird has high conservation value for conservationists and local people because of its cultural importance and also ecological role in vegetation regeneration (Tabiee and Tofighi 2017); thus, we recommend that the highly suitable habitats that are not currently protected be prioritized for conservation via current protected areas boundary expansion and new protected area designation. Our study provides the first country‐wide, fine‐resolution habitat suitability map for *P. pleskei* . We highlighted that while the species' potential distribution is broader than previously documented, many core habitats with high suitability remain outside current protected areas. Our results showed a significant conservation gap (64% of the suitable habitats of the species) that may undermine long‐term population viability, especially considering increasing anthropogenic threats (e.g., overgrazing and habitat destruction). We recommend that conservation authorities use these habitat suitability maps to prioritize unprotected high‐suitability zones for formal protection or community‐based conservation initiatives. Strengthening connectivity between fragmented habitats, preventing further habitat degradation, and raising local awareness are also essential steps toward effective species conservation.

## Author Contributions

**Masoud Yousefi:** conceptualization (equal), formal analysis (equal), investigation (equal), methodology (equal), software (equal), validation (equal), visualization (equal), writing – original draft (equal), writing – review and editing (equal). **Bagher Nezami:** formal analysis (equal), methodology (equal), visualization (equal), writing – original draft (equal), writing – review and editing (equal). **Faham Asgari:** data curation (equal), investigation (equal), methodology (equal), visualization (equal), writing – original draft (equal), writing – review and editing (equal). **Sayyad Sheykhi Illanloo:** data curation (equal), formal analysis (equal), validation (equal), visualization (equal), writing – original draft (equal), writing – review and editing (equal). **Sayedeh Alemohammad:** conceptualization (equal), data curation (equal), investigation (equal), visualization (equal), writing – original draft (equal), writing – review and editing (equal). **Luciano Bosso:** methodology (equal), supervision (lead), visualization (equal), writing – original draft (lead), writing – review and editing (lead).

## Funding

This work was supported by the National Recovery and Resilience Plan, CN_00000033. Iran National Science Foundation, 4032123.

## Conflicts of Interest

The authors declare no conflicts of interest.

## Supporting information


**Figure S1:** Protected areas network and elevation in Iran (Department of Environment of Iran, 2022).
**Table S1:** Records of *Podoces pleskei* used in species distribution models.

## Data Availability

All data supporting the findings of this study are provided in the Supporting Information or are freely accessible through the links referenced in the main text.
