# Supplementary material for: Climate Change Reduces Habitat Suitability of the Endemic Iranian Ground‐Jay (Podoces pleskei): Spatial Analyses to Guide Conservation Strategies
Source: Ecol Evol. 2026 Jun 9;16(6):e73637. doi: 10.1002/ece3.73637 (PMC13249540; doi:10.1002/ece3.73637)
Supplement: Supplementary file 1 — Figure S1: Protected areas network and elevation in Iran (Department of Environment of Iran, 2022). Table S1: Records of Podoces pleskei used in species distribution models. [file ECE3-16-e73637-s001.docx]

Supplementary materials

TABLE S1. Records of *Podoces pleskei* used in species distribution models.

| Species | Longitude | Latitude |
| --- | --- | --- |
| *Podoces pleskei* | 52.4368 | 34.49157 |
| *Podoces pleskei* | 52.88695 | 29.93441 |
| *Podoces pleskei* | 53.16915 | 30.19336 |
| *Podoces pleskei* | 53.48391 | 33.49535 |
| *Podoces pleskei* | 53.5445 | 32.02038 |
| *Podoces pleskei* | 54.2061 | 30.663 |
| *Podoces pleskei* | 54.20792 | 30.92932 |
| *Podoces pleskei* | 54.2131 | 30.65957 |
| *Podoces pleskei* | 54.2165 | 30.90507 |
| *Podoces pleskei* | 54.21653 | 30.90553 |
| *Podoces pleskei* | 54.2186 | 30.93963 |
| *Podoces pleskei* | 54.22639 | 31.13985 |
| *Podoces pleskei* | 54.23591 | 31.15678 |
| *Podoces pleskei* | 54.27394 | 31.3094 |
| *Podoces pleskei* | 54.30749 | 33.64108 |
| *Podoces pleskei* | 54.31 | 33.2 |
| *Podoces pleskei* | 54.43202 | 33.5334 |
| *Podoces pleskei* | 54.43609 | 33.56401 |
| *Podoces pleskei* | 54.44038 | 35.58619 |
| *Podoces pleskei* | 54.44557 | 33.40495 |
| *Podoces pleskei* | 54.45344 | 35.47377 |
| *Podoces pleskei* | 54.48579 | 35.57856 |
| *Podoces pleskei* | 54.62972 | 29.23534 |
| *Podoces pleskei* | 54.65638 | 29.3324 |
| *Podoces pleskei* | 54.82384 | 29.16625 |
| *Podoces pleskei* | 54.87438 | 30.12973 |
| *Podoces pleskei* | 54.88976 | 30.1194 |
| *Podoces pleskei* | 54.8899 | 30.1134 |
| *Podoces pleskei* | 54.8946 | 30.1083 |
| *Podoces pleskei* | 54.89548 | 30.14636 |
| *Podoces pleskei* | 54.8959 | 30.1356 |
| *Podoces pleskei* | 54.91877 | 30.15299 |
| *Podoces pleskei* | 54.9968 | 29.19255 |
| *Podoces pleskei* | 55.01 | 36.25 |
| *Podoces pleskei* | 55.00994 | 35.42741 |
| *Podoces pleskei* | 55.01243 | 36.26023 |
| *Podoces pleskei* | 55.0128 | 35.6251 |
| *Podoces pleskei* | 55.01358 | 35.56367 |
| *Podoces pleskei* | 55.02121 | 35.4342 |
| *Podoces pleskei* | 55.02369 | 35.42559 |
| *Podoces pleskei* | 55.04934 | 28.99497 |
| *Podoces pleskei* | 55.05714 | 35.71465 |
| *Podoces pleskei* | 55.05717 | 35.71502 |
| *Podoces pleskei* | 55.0577 | 35.64472 |
| *Podoces pleskei* | 55.1145 | 30.1235 |
| *Podoces pleskei* | 55.35879 | 36.2207 |
| *Podoces pleskei* | 55.42918 | 35.88535 |
| *Podoces pleskei* | 55.5325 | 30.53334 |
| *Podoces pleskei* | 55.5421 | 36.7888 |
| *Podoces pleskei* | 55.59354 | 36.05026 |
| *Podoces pleskei* | 55.62477 | 36.71326 |
| *Podoces pleskei* | 55.63477 | 35.82672 |
| *Podoces pleskei* | 55.669 | 36.7078 |
| *Podoces pleskei* | 55.7473 | 35.9277 |
| *Podoces pleskei* | 55.79784 | 35.79721 |
| *Podoces pleskei* | 55.8005 | 35.7964 |
| *Podoces pleskei* | 55.83917 | 35.90155 |
| *Podoces pleskei* | 55.88339 | 35.9766 |
| *Podoces pleskei* | 55.93589 | 35.6418 |
| *Podoces pleskei* | 55.9441 | 36.03466 |
| *Podoces pleskei* | 55.96965 | 36.02978 |
| *Podoces pleskei* | 56.00261 | 35.99904 |
| *Podoces pleskei* | 56.01667 | 35.90193 |
| *Podoces pleskei* | 56.02486 | 35.85439 |
| *Podoces pleskei* | 56.03515 | 35.96455 |
| *Podoces pleskei* | 56.04293 | 35.78952 |
| *Podoces pleskei* | 56.04489 | 35.86563 |
| *Podoces pleskei* | 56.04654 | 35.94809 |
| *Podoces pleskei* | 56.04881 | 36.08807 |
| *Podoces pleskei* | 56.06475 | 35.96797 |
| *Podoces pleskei* | 56.0658 | 35.96681 |
| *Podoces pleskei* | 56.06601 | 35.96654 |
| *Podoces pleskei* | 56.06733 | 35.96637 |
| *Podoces pleskei* | 56.06758 | 35.96649 |
| *Podoces pleskei* | 56.06868 | 35.95943 |
| *Podoces pleskei* | 56.07 | 35.9432 |
| *Podoces pleskei* | 56.08014 | 35.70219 |
| *Podoces pleskei* | 56.08285 | 35.74424 |
| *Podoces pleskei* | 56.08391 | 36.58504 |
| *Podoces pleskei* | 56.09158 | 35.97813 |
| *Podoces pleskei* | 56.09292 | 35.96341 |
| *Podoces pleskei* | 56.10001 | 35.70949 |
| *Podoces pleskei* | 56.10189 | 35.93294 |
| *Podoces pleskei* | 56.10346 | 35.9756 |
| *Podoces pleskei* | 56.10362 | 35.97399 |
| *Podoces pleskei* | 56.11042 | 35.72384 |
| *Podoces pleskei* | 56.11109 | 35.9412 |
| *Podoces pleskei* | 56.11297 | 35.96773 |
| *Podoces pleskei* | 56.11975 | 35.9138 |
| *Podoces pleskei* | 56.1199 | 35.95305 |
| *Podoces pleskei* | 56.12138 | 35.91372 |
| *Podoces pleskei* | 56.1224 | 35.9681 |
| *Podoces pleskei* | 56.14001 | 35.95868 |
| *Podoces pleskei* | 56.17717 | 35.91352 |
| *Podoces pleskei* | 56.17841 | 35.91302 |
| *Podoces pleskei* | 56.18736 | 35.91619 |
| *Podoces pleskei* | 56.19194 | 35.94674 |
| *Podoces pleskei* | 56.2004 | 35.81464 |
| *Podoces pleskei* | 56.21985 | 35.83151 |
| *Podoces pleskei* | 56.23612 | 35.92884 |
| *Podoces pleskei* | 56.33168 | 35.91697 |
| *Podoces pleskei* | 56.33664 | 35.88743 |
| *Podoces pleskei* | 56.37723 | 35.87539 |
| *Podoces pleskei* | 56.4598 | 35.8588 |
| *Podoces pleskei* | 56.51495 | 35.83634 |
| *Podoces pleskei* | 56.51495 | 35.83634 |
| *Podoces pleskei* | 56.58851 | 35.89102 |
| *Podoces pleskei* | 56.69589 | 30.08667 |
| *Podoces pleskei* | 57.05626 | 35.91446 |
| *Podoces pleskei* | 57.51785 | 33.29855 |
| *Podoces pleskei* | 59.62751 | 31.06409 |


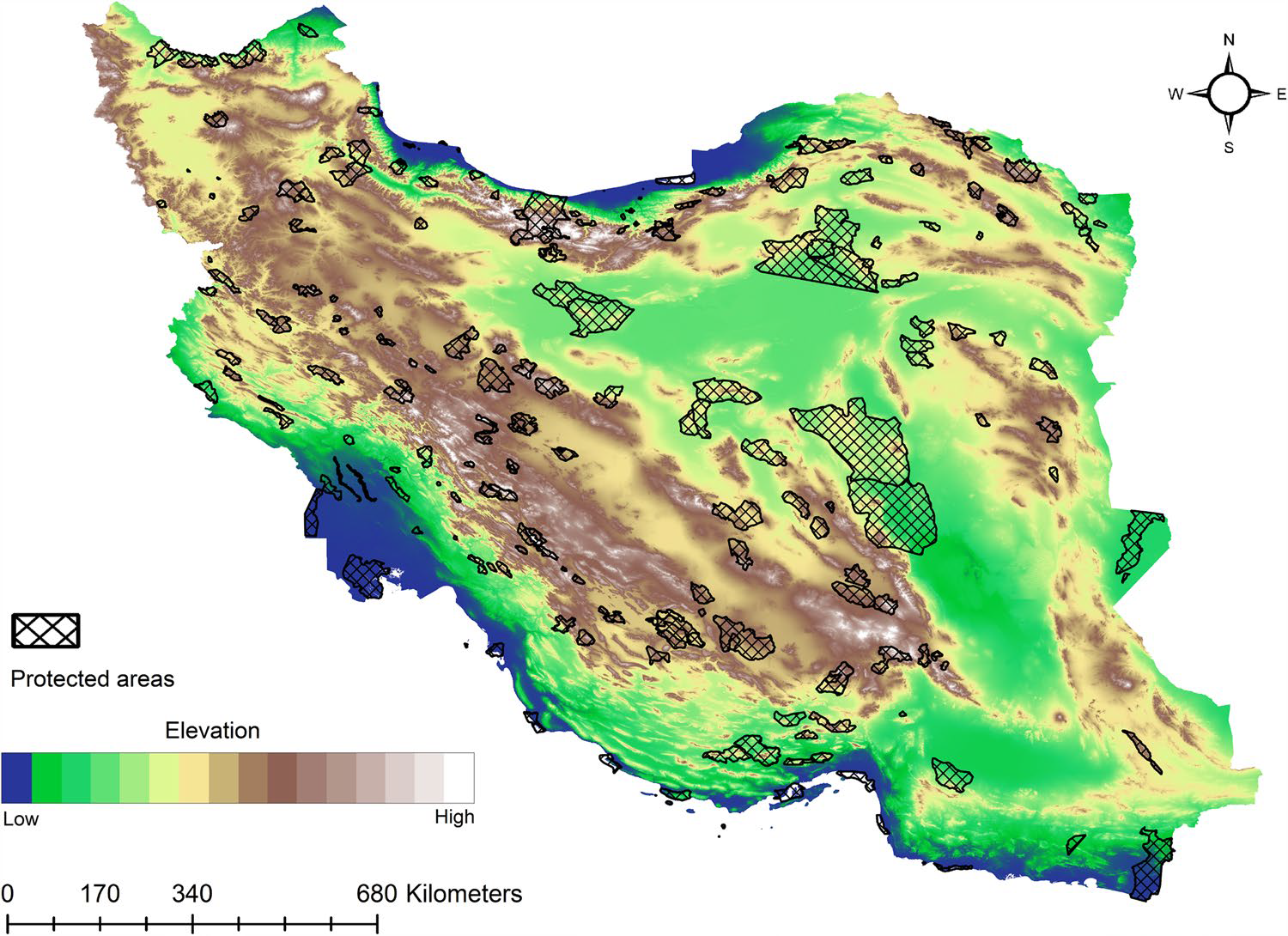


FIGURE S1. Protected areas network and elevation in Iran (Department of Environment of Iran, 2022).
